# Supplementary material for: Involvement of CaV2.2 channels and α2δ‐1 in homeostatic synaptic plasticity in cultured hippocampal neurons
Source: J Physiol. 2022 Dec 3;600(24):5333–51. doi: 10.1113/JP283600 (PMC10107484; doi:10.1113/JP283600)

Journal of Physiology

Original Western Blots

For manuscript #JP-RP-2022-283600R1

Fig 1 D P1

$Ca_V2.2$_HA | GAPDH

Experiment 1

Experiment 2

Experiment 3

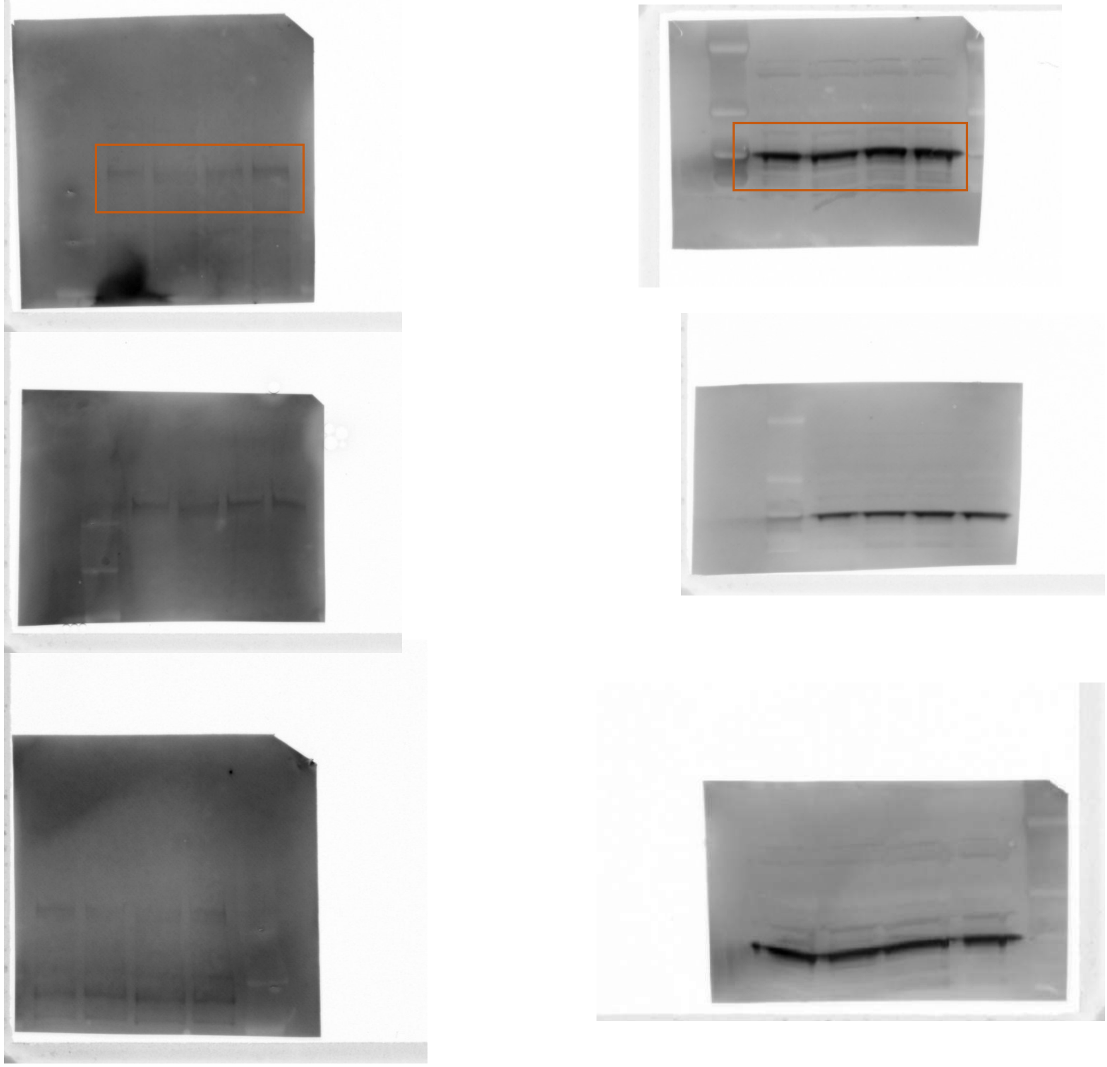

Lanes used as representative for figure

Fig 1 E P7

Experiment 1

Experiment 2

Experiment 3

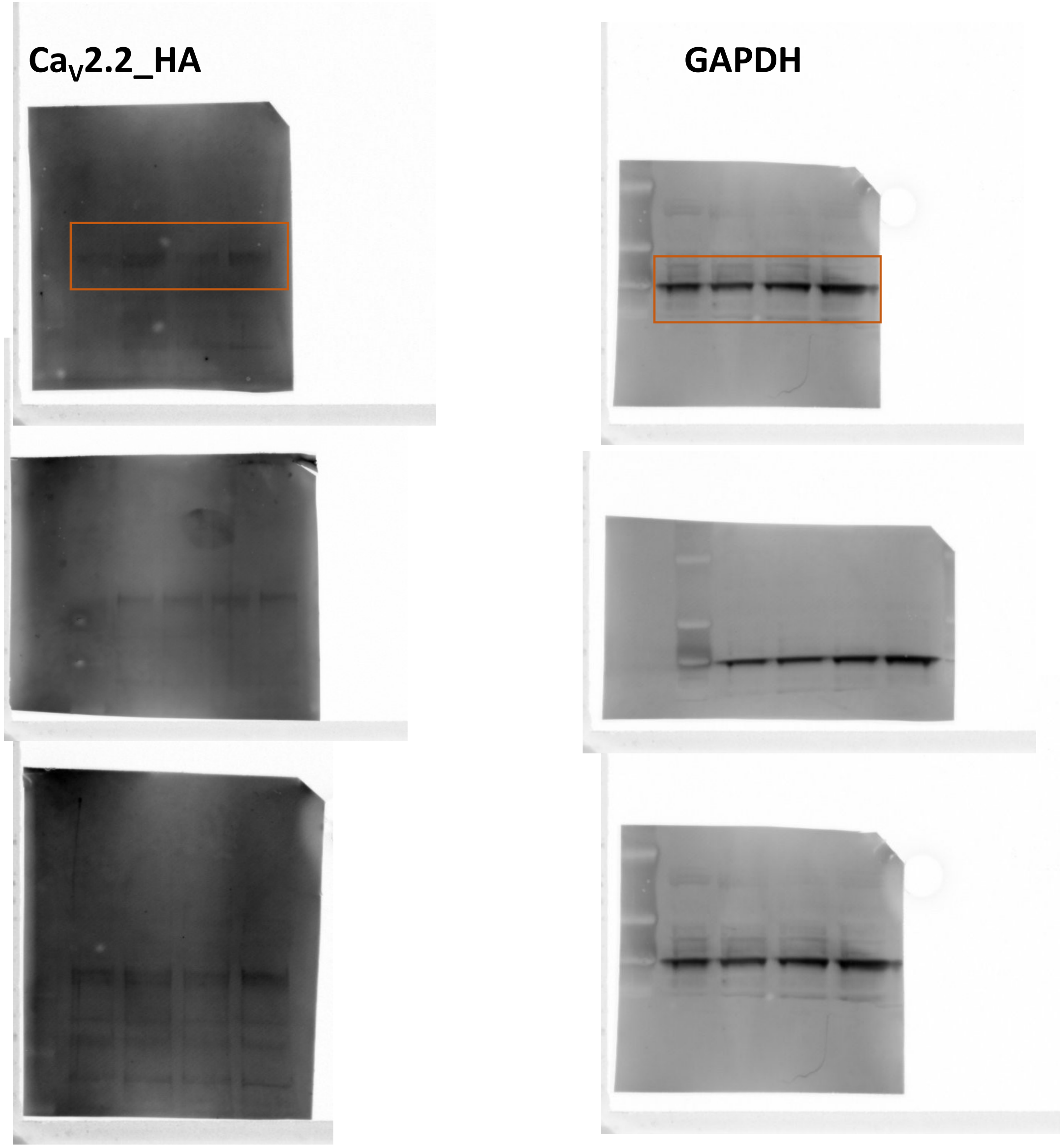

Lanes used as representative for figure

Fig 1 F 12 weeks

Ca_V2.2_HA | **GAPDH**

Experiment 1

Experiment 2

Experiment 3

Experiment 4

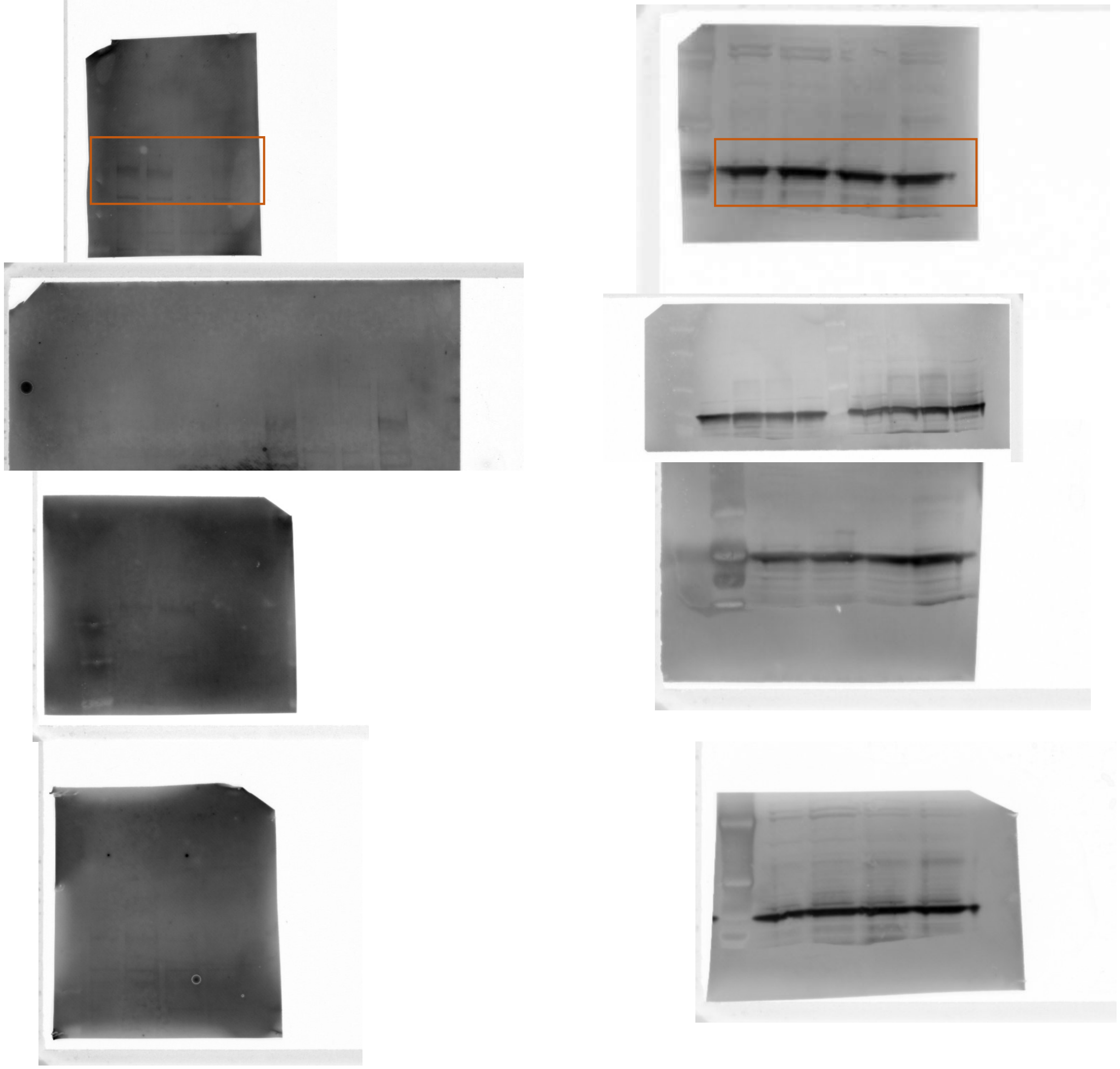

Lanes used as representative for figure

Fig 4 Whole cell lysates

Ca$_V$2.2 II-III loop

GAPDH

Experiment 1

Experiment 2

Lanes used as representative for figure

Experiment 3

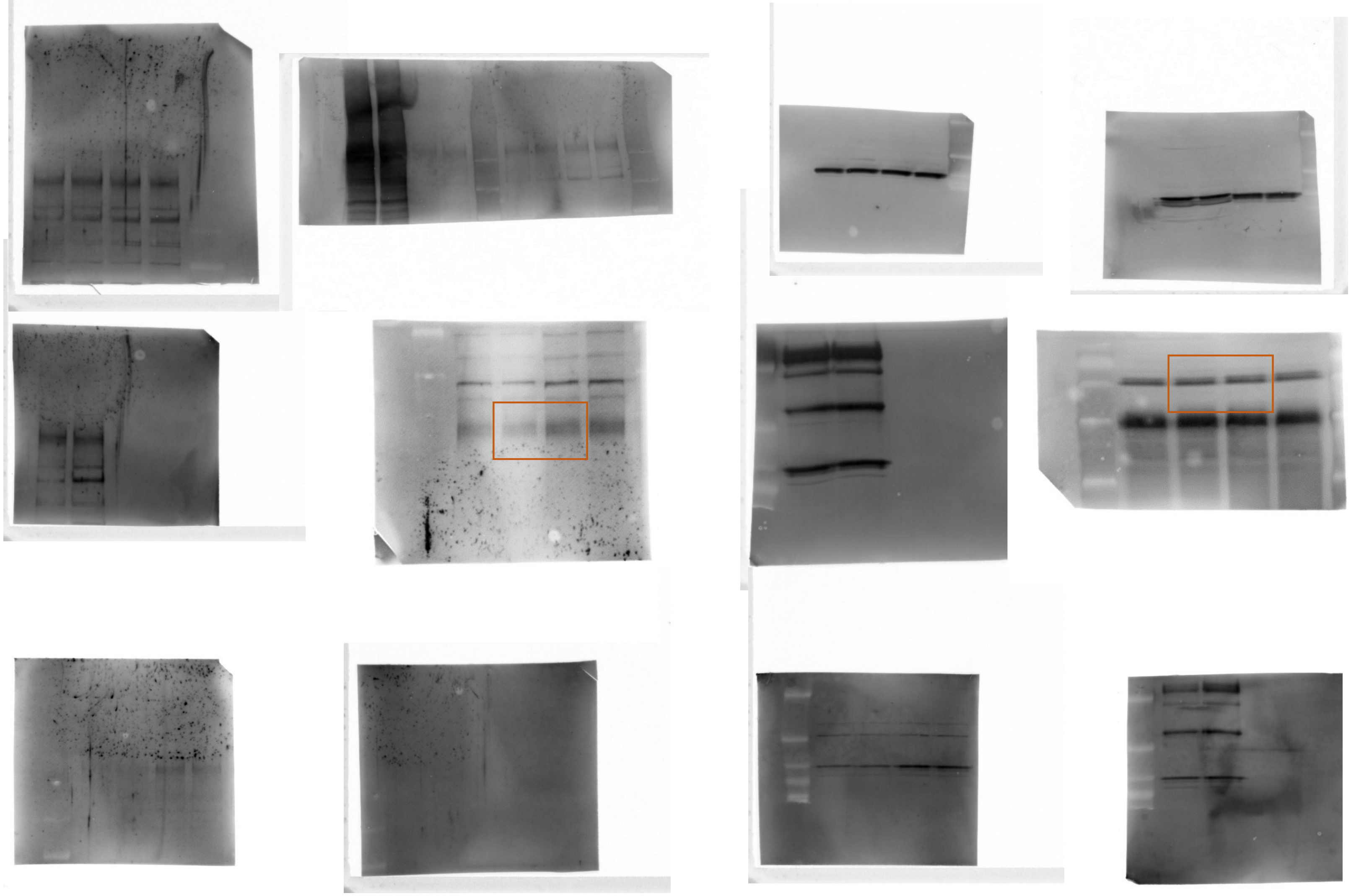

Fig 6 A Biotin experiments

A2δ-1 Ab

GAPDH

Experiment 1

Lanes used as representative for figure

Experiment 2

Experiment 3
Only WCL used

Experiment 4

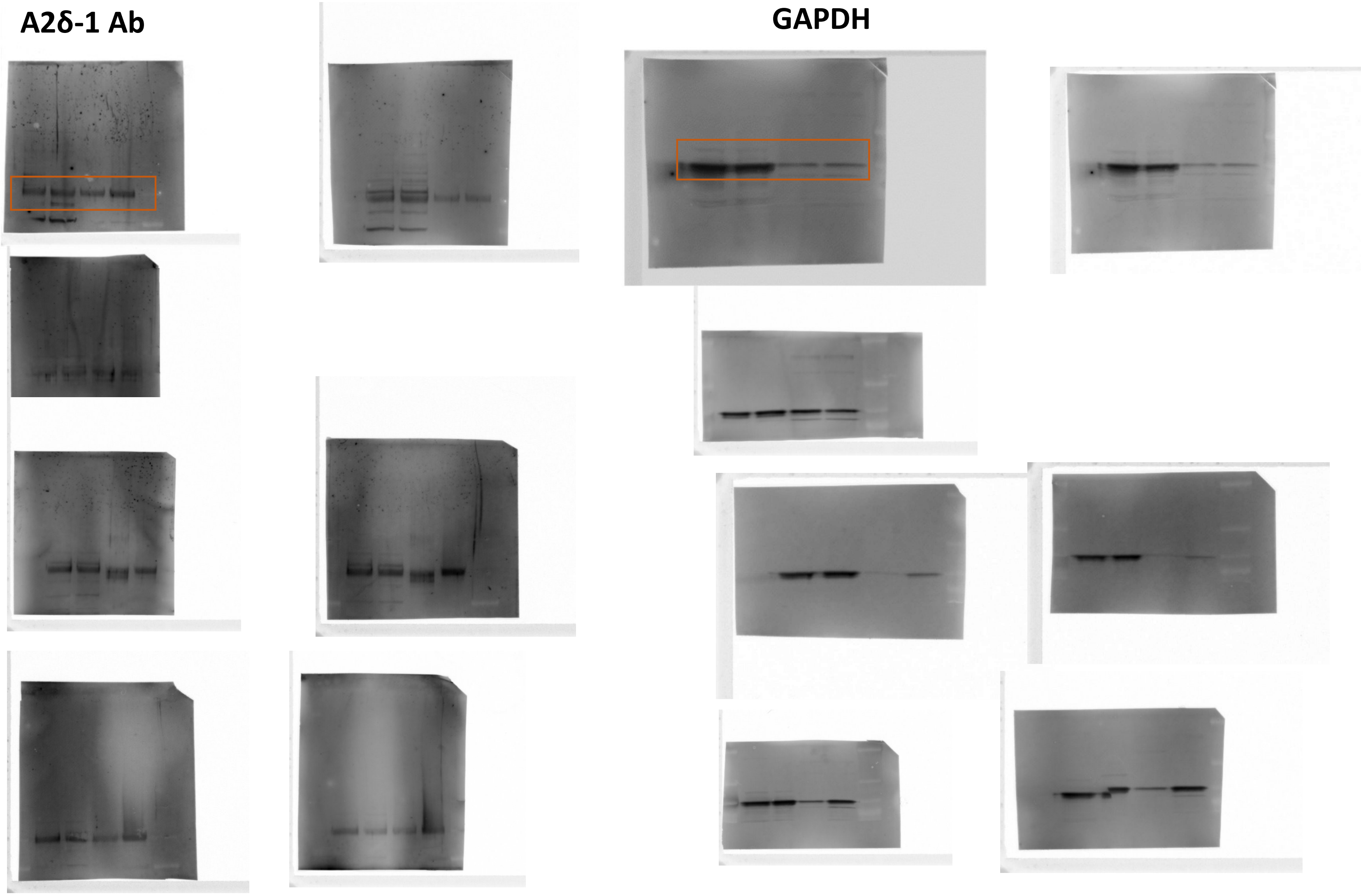

Supplement: Supplementary file 3 — Original western blots [file TJP-600-5333-s004.pdf]
